# Heavy Foil

**Published:** 1870-03

**Authors:** 


### SELECTED ARTICLES.

### ARTICLE V.

### *Heavy Foil.*

The latest novelty, in dental science, attracting much notice, is the use of heavy foil. Foils ranging from 20 to 120 grains to the sheet. The credit of introducing this new sensation, is due, so far as we have been able to learn, to Dr. W. H. Atkinson, of New York. It was brought to our notice by Dr. H. S. McKellops of this city. Dr. McK., while on a visit to New York the past summer, called upon Dr. A. who, with his usual urbanity and politeness, invited his friend to witness the operation of filling teeth with the heavy foils, Nos. 15, 20, 30. Dr. McK. reports himself as very much surprised at the apparent ease with which the Dr. manipulated them in all classes of cavities, and at the fine results produced. So well pleased was he with what he saw, that immediately on his return home, he commenced the use of heavy foil in his own practice. The attention of ourself, and other members of the profession, in the city, was called to it by Dr. Mc., who invited us to witness the operation of filling with the heavy Nos., 20, 30, 60. The result has been the general—I might say the exclusive adoption of them by all who have given them a fair trial. Prof. Judd is using No. 120, in some special cases, and speaks of it in the highest terms, but thinks Nos. 20 and 30 will be most generally used; uses 120 on large flat surfaces.

Having adopted the use of them ourself, to the exclusion of all the lower Nos., and being daily better pleased with the results, and most fully satisfied that it is the best form of gold for filling extant, we ask the attention of our readers to a few facts respecting heavy foils:

Whew! says one, what a piece of nonsense. You are certainly not going to ask the profession to adopt this new folly because Drs. A. and B. see fit to recommend it. Why, the ink is hardly dry on your letter recommending the extreme low Nos., 2 and 3, for the same advantages now claimed for Nos. 20, 30, 60. What inconsistency! Do you suppose the profession will accept and adopt, as new and practical, a form of gold, which was tried long years ago, by one of the best operators in the profession, Dr. Arthur, and given up as impracticable. Because *some men* stir the broth must we drink it? Now, dear reader, what matters it who does the stirring if the broth be drinkable? If we are not able to do the stirring, don't let us deny others the privilege, or refuse to drink because we had no hand in the making. Having tasted this last bowl of broth, we are prepared to recommend it; we know its components and the principle upon which it is concocted; we know it to be savory, and believe it to be a promoter of health. We are willing to acknowledge it does look a little inconsistent, after what has been said in favor of No. 2. Still we think, a pretty strong case can be made for No. 60. I am also aware that Dr. Arthur tried heavy foil, but I am not disposed to allow that the foil he used possessed the same qualities as that we now have. Neither was his mode of manipulation the same as we now propose.

We adopted the use of No. 2, and recommended it for its apparent softness, adhesiveness, and for the ease with which it was manipulated; we say apparent, because we now believe it does not possess in fact, these properties in excess, only in appearance. Being so extremely thin it offered but little resistance to the instrument, and the particles were so easily driven together, and so firmly held by the mechanical process of interlocking that we were led to believe it possessed all the requisites of a perfect filling. If we but study the properties of this metal, gold, and note some of the laws governing it, we may be able to account for some of the advantages claimed for No 60. Pure gold is a soft malleable ductile, tenacious metal, crystalline or cellular in structure; specific gravity 19.2, which may be increased to 10.6+ by lamination. It possesses the pecular property of adhesiveness, or cold welding. By lamination the malleability, ductility and tenacity, as well as density is increased to a limited degree, while it is rendered less soft, and the cold welding property is perceptibly diminished.

A trace of iron, copper, or silver, as an alloy, makes it harder, and perceptibly lessens its adhesive property, while the other properties mentioned are increased. The molecular condition of the surfaces as well as of the mass affect the adhesiveness. Foil when taken from the cutch is hard, elastic and non-adhesive. Change its molecular condition by annealing, and it becomes non-elastic and adhesive, dependent upon the degree of heat to which it is subjected. Foil subjected to the action of certain gases, as sulphuretted hydrogen and phosphoric acid, at once loses its adhesive property, while ammoniacal gas works another change, rendering it more soft and velvety. We have at the present time several forms of this metal, in which it is used for the purposes of filling teeth, viz: sponge or crystal and fibrous gold, (all similar in character). Non-adhesive soft foils, ranging in Nos. from 4 to 10 grs. to the sheet, adhesive foils in Nos. from 2 to 6, and last, but not least, adhesive foils in Nos. from 20 to 120. If we compare these different forms with one which shall posses all the requisites of a perfect filling, being aided in our comparison by the known properties and laws governing the metal, we may place a very fair estimate on the comparative value of each. For instance, with the present mode of manipulating a filling by mallet pressure, the gold should be soft, tenacious, adhesive; readily adapted to the walls of the cavity, and capable of being rendered uniformly solid, without the expenditure of too much time or labor. If we compare sponge or crystal gold by this standard, we find it soft as desirable, but wanting in tenacity.

It is readily adapted to the walls of the cavity, but it requires too much time and labor to thoroughly condense it and to secure perfect margins. Non-adhesive foil is sufficiently soft and tenacious, and is quite readily adapted to the walls, but lacking the adhesive property it is not so well adapted to the present mode of manipulation, and to all classes of cases, as sponge or crystal gold. Adhesive foils, Nos. 2 to 6,* are harder than the sponge gold or the soft non-adhesive foils, harder also than the heavy Nos. of adhesive foil. The extreme attenuation of Nos. 2 and 3 makes them less tenacious and adhesive than the heavy Nos. Being hard and somewhat elastic, they are not so easily adapted to the walls as the non adhesive foils, and more time and care is required to properly condense them. Being exceedingly thin, and wanting in tenacity, they are easily broken and torn into small fragments or scales, which are not sufficiently adherent to stick to the face of a plug, and consequently break away in a short time, and are washed away by the fluids of the mouth ; hence we find the surface of a large majority of the plugs made with these low Nos. presenting a rough unfinished appearance, full of minute pits from the serrations of the plugger.

Adhesive foil Nos. 20 to 60 and upward, approaches more nearly the sponge or crystal form in softness, because in the process of annealing it is brought back to nearly the same molecular condition. It also regains the property of adhesiveness to a much greater degree than the low Nos. Having been rendered fibrous by lamination it excels the sponge or crystal in tenactiy, and it is therefore superior to these forms in that respect. It does not break up or crumble under the instrument as these forms are apt to do, If properly manipulated it is readily adapted to the walls of the cavity, and with the same expenditure of time and care it may be more thoroughly condensed, and better margins can be secured than with any other form ; being soft, non-elastic, tenacious, and adhesive, it may be carried over the edge of a frail wall without crumbling the wall in the least, its thickness preventing injury to the border or wall of the cavity by the sharp points of the plugger being driven through it, a difficulty attending the use of crystal gold, and the extreme low numbers of foil. Its extreme adhesiveness admits of the use of very fine serrations on the point of the plugger, hence the surface of the plug presents a more solid and uniform appearance: being very tenacious it makes a more lasting surface, because the foil is not broken or torn by the instrument in condensing, as has been observed is the case with the low Nos. and with crystal and sponge gold.

We have thus briefly endeavored to give our readers some of the principal reasons for our adopting this new hobby of Dr, A's., and we feel assured of receiving their thanks for thus calling attention to it, if they will but give it a fair trial. Our mode of manipulating the heavy foil may be the subject of another article.—*Missouri Dental Journal.*

